# Performance, Emission, Energy, and Exergy Analysis of a C.I. Engine Using Mahua Biodiesel Blends with Diesel

**DOI:** 10.1155/2014/207465

**Published:** 2014-10-29

**Authors:** Nabnit Panigrahi, Mahendra Kumar Mohanty, Sruti Ranjan Mishra, Ramesh Chandra Mohanty

**Affiliations:** ^1^Department of Mechanical Engineering, Gandhi Institute of Technology and Management, Bhubaneswar 752054, India; ^2^College of Agricultural Engineering and Technology, OUAT, Bhubaneswar 751003, India; ^3^Department of Chemistry, C.V. Raman College of Engineering, Janla, Bhubaneswar 752054, India; ^4^Department of Mechanical Engineering, Centurion University of Technology and Management, Khurda 752050, India

## Abstract

This paper presents an experimental investigation on a four-stroke single cylinder diesel engine fuelled with the blends of Mahua oil methyl ester (MOME) and diesel. The performance emission, energy, and exergy analysis has been carried out in B20 (mixture of 80% diesel by volume with 20% MOME). From energy analysis, it was observed that the fuel energy input as well as energy carried away by exhaust gases was 6.25% and 11.86% more in case of diesel than that of B20. The unaccounted losses were 10.21% more in case of diesel than B20. The energy efficiency was 28%, while the total losses were 72% for diesel. In case of B20, the efficiency was 65.74 % higher than that of diesel. The exergy analysis shows that the input availability of diesel fuel is 1.46% more than that of B20. For availability in brake power as well as exhaust gases of diesel were 5.66 and 32% more than that of B20. Destructed availability of B20 was 0.97% more than diesel. Thus, as per as performance, emission, energy, and exergy part were concerned; B20 is found to be very close with that of diesel.

## 1. Introduction

The consumption of petroleum products in India is 150 million metric tons per year. Primary commercial energy demand growth is 5% per year. India accounted for 3.9% of the world's commercial energy demand (Infraline Energy Report.). India's growing dependence on imported oil products and the domestic rise in the crude oil prices have recently been of great concern which affects the country's economy and development. Pollution also remains a major challenge. Air pollution is a serious issue with the major sources being vehicle emission. These factors have compelled the researchers to find an alternative solution. In recent years in the context of climate changes and of soaring prices for diesel, biodiesel is now being presented as a renewable alternative energy to petro-diesel by different researchers. 7% of total renewable energy is available in wide forms and sources.

Currently in many countries, the emissions of diesel engines running on petrodiesel are strictly regulated. The upper limits for the emission of CO, CO_2_, NO_*x*_, THC (total unburned hydrocarbons), and PM (particulate matters) have been defined. These limits and scarcity of petroleum resources have promoted researchers to go for an alternative fuel used in C.I. engines.

Experiments study shows that the use of pure oils (SVO = straight vegetable oil or PPO = pure plant oil) is becoming of interest as alternative fuels for diesel engines. This is especially a case in remote areas in developing countries, where petrodiesel and biodiesel are often not readily available or expensive [2]. For engines designed to burn diesel fuel, the viscosity of vegetable oil must be lowered to allow for proper atomization of the fuel; otherwise, incomplete combustion and carbon buildup will ultimately damage the engine (vegetable oil fuel from Wikipedia, the free encyclopedia). Principally, the viscosity and surface tension of SVO/PPO must be reduced by preheating it, typically by using waste heat from the engine or electricity; otherwise, poor atomization, incomplete combustion, and carbonization may result. One common solution is to add a heat exchanger and an additional fuel tank for the petrodiesel or biodiesel blend and to get switch between this additional tank and main tank of SVO/PPO which is expensive (modified fuel system by Wikipedia, the free encyclopedia). During the preheated SVO, engine decreases the power and efficiency [3].

Conclusions were drawn by researcher based on experiments study: Navindgi et al. [4] have carried out the experiments with preheated SVO of Neem, Mahua, and Castor and found that neat oil with preheating can be substituted as fuel for diesel engine. Kapilan et al. [5] used Mahua oil as fuel in the diesel engine and concluded that the thermal efficiency is found to be lower while smoke emission is found to be higher. Acharya et al. (2011) [6] have conducted an experiment on preheated SVO up to 130°C in order to reduce viscosity. The result obtained was that more preheated SVO oil was needed to produce the same amount of energy produced by the pure diesel fuel. Regarding the brake thermal efficiency of preheated SVO, it was lower than that of diesel throughout the entire range. Effect of exhaust mission also shows poor result. HC, CO, CO_2_, NO_*x*_, and smoke composition were found much higher in comparison to diesel. These problems arise due to high viscosity, low volatility character, and increased combustion temperature of oxygenated fuel. The fuel injection system of new technologies engines is sensitive to fuel viscosity changes. High viscosity of oil which is due to high free fatty acid (FFA) may lead to poor combustion, injector chocking, ring sticking, injector deposits, and injector pump failure. Sahoo et al. [7] concluded that transesterification is one of the most reliable and commonly used techniques to produce biodiesel from oil seeds.

Energy analysis studies show that 1/3 rd of the energy of a fossil fuel is destroyed during the combustion process [8]. Palm oil methyl ester (POME) run engine can recover around 26% of the energy supplied by the fuel [9]. Soybean biodiesel shows similar energetic performance values with that of petroleum diesel fuel [10]. Thermal efficiency of engine fuelled by diesel was slightly higher than B50 palm oil biodiesel [11].

In this study, experiments were performed on a single cylinder, four-stroke, 3.5 Kw diesel engine at various capacities of the engine. The engine was first operated by diesel and then followed by various blends of MOME. The performance and emission characteristics were investigated. For energy analysis, the 1st law of thermodynamics is applied to quantify various losses of the above engine by using diesel fuel and B20 at full load. For exergy analysis, the 2nd law of thermodynamics is applied to determine the available work of a four-stroke diesel engine by using diesel and B20.

*Madhuca longifolia,* commonly known as Mahua, is an Indian tropical tree found largely in the central and north Indian plains and forests. It is a fast-growing tree and grows to approximately 20 metres in height, possesses evergreen or semievergreen foliage, and belongs to the family Sapotaceae. It is adapted to arid environments, being a prominent tree in tropical mixed deciduous forests in India in the states of Chhattisgarh, Jharkhand, UP, MP, and Bihar and Odisha. It is cultivated in warm and humid regions producing between 20 and 200 kg of seeds annually per tree depending on maturity (*Madhuca longifolia*, Wikipedia.). Flowers are cream colored, corollas fleshy, and juicy and clustered at the end of branches. Fruits are berries, ovoid, fleshy, turning, yellowish green when ripe, and 3–5 cm long. Seed is large, 3-4 cm long, and elliptical on one side [12].

**Figure 1:**
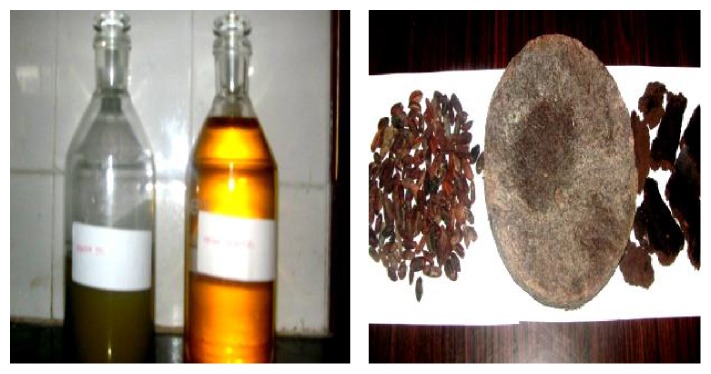
Mahua oil and biodiesel, seeds, and cakes.

In villages, oil extraction is done by local method, after the flowers that ripen, known as tola (local name), are collected. It is then put in a water container so that it will be easy to obtain seeds from kernels. Kernels are separated to obtain the seeds. These seeds are then turned into small pieces followed by drying these small pieces seeds in hot atmospheric condition for 2 to 3 hours. The oil yields are done by local ghanis. The Mahua oil obtained by pressing is collected in a drum. Thus, filtration is done to remove the various unwanted particles left in the extracted oil in order to obtain the pure Mahua oil. The oil yields from ghanis are 20–30%, while those of oil expellers are 34–37%, respectively. The expelled cake was relevant to recover the residual oil and after that cakes were used as fertilizers for agriculture purpose. Fresh oil from properly stored seeds is yellow, while commercial oils are generally greenish yellow with disagreeable odor and taste. FFA oil extracted from fresh kernels are less than 1-2% compared to stored extracted kernels which is around 30% (biodiesel business: prospect for profitable sustainability). Mahua seeds, oil, biodiesel, and cakes are shown in Figure 1.

Mahua seed contains 35% oil and 16% protein. The fatty acid profile of Mahua oil is shown in Table 1.

## 2. Materials and Methods

### 2.1. Oil Extraction

**Figure 2:**
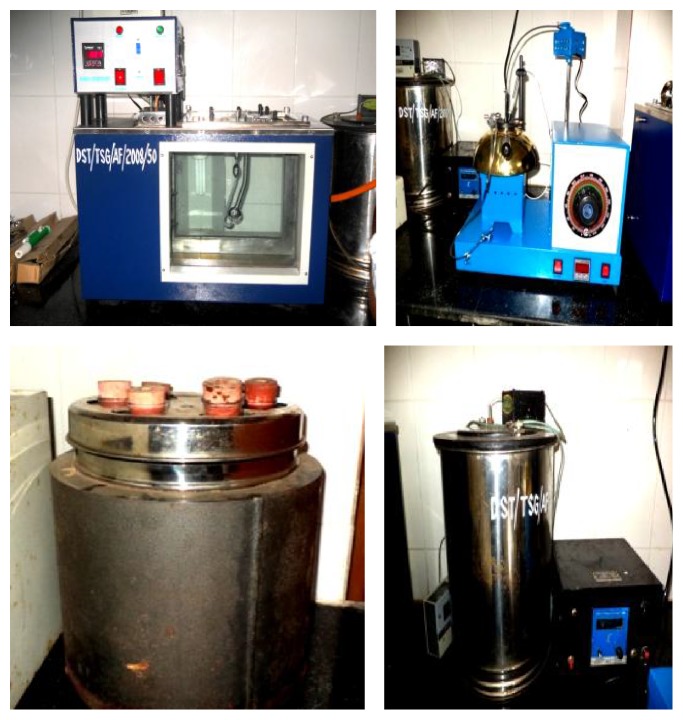
Apparatus used in renewable energy lab.

Crude Mahua oil was purchased from karanji village at keonjhar district. Commercial diesel was purchased from nearby Indian oil filling station. The oil experiment was carried out in the Renewable Energy Lab of Orissa University of Agriculture and Technology (OUAT), Bhubaneswar, Odisha. Various apparatus in this lab are shown in Figure 2. All chemicals like methanol, acid catalyst, sulphuric acid (H_2_SO_4_), alkali catalyst (KOH), and so forth, used of analytical grade, were available in the lab. The acid value of crude Mahua oil was found to be 7.84 which was greater than 6. To achieve maximum conversion of biodiesel from high FFA oil two steps (esterification and transesterification) methods were used [13].

### 2.2. Acid Method

Under this process, the acid catalyst mixture {12.5 mL  of  H_2_SO_4_  (1%) + 250 mL  methanol  (20%)} was added to 1250 mL. preheated oil (oil was heated at 60°C for 15 minutes) samples. The reactants are stirred at a speed of 1600 rpm at a temperature of 60°C for 2 hours resulting in reduction of the acid value; that is, the FFA was reduced to 2.8. After the reaction, the lower glycerol layer was decanted and the upper ester layer was taken for transesterification. Once the reaction was complete, it was dewatered by passing it over anhydrous Na_2_SO_4_ before transesterification.

### 2.3. Alkali Catalyzed Method

The above sample was heated and chemicals {6  gram  of  KOH  (0.5%) + 250 mL  methanol  (20%)} were added. A part of the KOH was used to neutralize the residual amount of acid and the remaining KOH was used for transesterification.

### 2.4. Purification and Drying

The product was allowed to stand overnight to separate the biodiesel and glycerol layer. The upper biodiesel layer was separated from the glycerol layer and washed with hot distilled water to remove the excess methanol, catalyst, and traces of glycerol. The washed ester layer was dried at 70°C under the vacuum to remove the moisture and methanol and again passed over anhydrous Na_2_SO_4_. The biodiesel obtained was designated by MOME having acid value of 1.12 [14]. The acid value of Mahua oil is shown in Table 2. Fuel properties of Mahua Oil, MOME, and Diesel are given in Table 3.

### 2.5. Experimental Engine Setup

**Figure 3:**
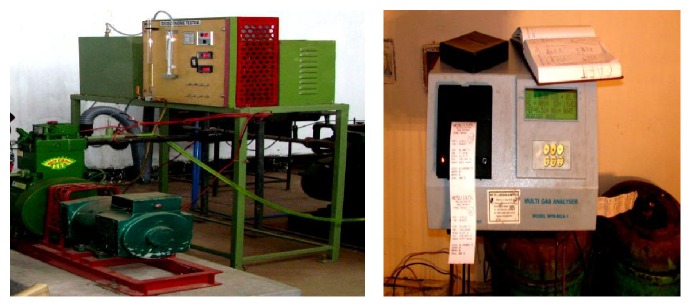
Experimental setup showing the diesel engine and multigas analyzer.

Experiments were performed in the Engine Testing Lab, OUAT, Bhubaneswar, on a single cylinder four-stroke diesel engine by using MOME. The performance and emission characteristics were investigated. The engine was coupled with a single-phase 230 V AC alternator with electrical loading of different loads in watts. A multigas analyzer (Model NPM MGM-1) made by Netel (India) Pvt limited was used for various exhaust gas emissions. The engine was first operated by diesel and the correspondence readings were taken and then followed by various blends of MOME. Experimental setup is shown in Figure 3.

The specifications of the engine are shown in Table 4.

### 2.6. Energy Analysis

An energy analysis sheet is an account of energy supplied and utilized by using diesel and B20. Reference atmospheric conditions are considered as 1 atm. (*P*_atm_) and 27°C (*T*_amb_). For the purpose of analysis of the 1st law of thermodynamics, the following assumptions are made.

1. The engine runs at a steady state.
2. The whole system is selected as a control volume.
3. The composition of air and exhaust gas each forms ideal gas mixtures.
4. Potential and Kinetic energy effects of the incoming and outgoing fluid streams are ignored.

The fuel energy supplied to the engine is in the form of fuel heat. The various ways in which this fuel energy is used in the system are heat equivalent of brake power, energy carried away by cooling water, and energy carried away by the exhaust gasses.

#### 2.6.1. Energy Balance

1st law of thermodynamics can be expressed as “the net change (increase or decrease) in the total energy of the system during a process is equal to the difference between the total energy entering and the total energy leaving the system during that process.”

That is, total energy entering the system − total energy leaving the system = change in the total energy of the system.

For steady-flow system,

(1) $$\begin{array}{c} {\dot{E}}_{\text{in}}={\dot{E}}_{\text{out}}. \end{array}$$

That is, rate of net energy transfer by heat, work, and mass = rate of change in internal, kinetic, potential, and so forth energies [15]. This relation is referred to as the* energy balance* and is applicable to any kind of system and any kind of process. Therefore, a general steady flow system can be written as

(2) Q.in+W.in+∑inmh+V22+gz  =Q.out+W.out+∑outmh+V22+gz.

(3) $$\begin{array}{c} \text{or},\;\;\overset{\cdot }{Q}-\overset{\cdot }{W}=\dot{m}\left[h_{2}-h_{1}+\frac{V_{2}^{2}}{2}-\frac{{\dot{V}}_{1}^{2}}{2}+g\left(z_{2}-z_{1}\right)\right], \end{array}$$

where $\dot{Q}$ is the heat transfer, $\dot{W}$ is the work done, $\dot{m}$ is the mass transfer, *h* is the enthalpy, *V* is the velocity, *Z* is the elevation, and *t* is the time taken. Obtaining a negative quantity for *Q* or *W* simply means that the assumed direction is wrong and should be reversed [15].

Neglecting the potential energy and kinetic energy, (3) can be written on unit-mass basis as

(4) q−w=m.h2−h1=m.Δh=m.∫12CpTdT=m.CpT2−T1,

where $q=\dot{Q}/\dot{m}$ is the heat transfer per unit mass. $w=\dot{W}/\dot{m}$ is the work done per unit mass. *T* is the corresponding temperature. *C*_*p*_ is the specific heat at constant pressure.

Applying (4) to any heat exchanger device, the equation is reduced to

(5) $$\begin{array}{c} {\dot{Q}}_{w,\text{in}}={\dot{m}}_{w}\left(h_{2}-h_{1}\right)={\dot{m}}_{w}C_{p}\left(T_{2}-T_{1}\right). \end{array}$$

#### 2.6.2. Energy Balance Calculations for the Present Experiment

An energy balance or heat balance sheet is an account of heat supplied and heat utilized in various ways in the engine. The sequence of events in the engine are fuel and air combustion, conversion of chemical energy to mechanical work, heat loss through cooling water to cool the engine head, and heat loss by the exhaust gas through calorimeter.

The heat supplied to the engine is only in the form of fuel heat. (*Q*_*s*_) in kW:

(6) $$\begin{array}{c} Q_{s}=m_{f}\times \text{L}.\text{C}.\text{V}., \end{array}$$

where *m*_*f*_ is the mass of fuel supplied in kg/sec. L.C.V. is the lower calorific value of the fuel in kJ/kg.

The various ways in which heat is used in the engine system is given by the following.

*(i) Heat Equivalent of Brake Power *(*Q*_*BP*_)* in kW, is*

(7) $$\begin{array}{c} Q_{\text{BP}}=2\times \pi \times N\times T_{e}, \end{array}$$

where *N* is the crank revolution per second. *T*_*e*_ is the torque developed in kN·m.

*(ii) Heat Carried Away by Cooling Water *(*Q*_*cw*_)* in kW*. Consider

(8) $$\begin{array}{c} Q_{\text{cw}}={m_{we}\times C}_{pw}\times \left(T_{2}-T_{1}\right), \end{array}$$

where *m*_*we*_ is the mass of cooling water circulated through the cooling jacket in kg/sec. *C*_*pw*_ is the specific heat of water in kJ/kg·K. *T*_2_ − *T*_1_ is the rise in temperature of the water passing through the cooling jacket of the engine in K.

*(iii) Heat Carried Away by Exhaust Gases *(*Q*_*ex*_)* in kW*. Consider

(9) $$\begin{array}{c} Q_{\text{ex}}=m_{ge}\times C_{pe}\times \left(T_{5}-T_{a}\right), \end{array}$$

where *m*_*ge*_ is the mass of exhaust gases (*m*_*f*_ + *m*_*a*_) in Kg/sec. *C*_*pe*_ is the specific heat of exhaust gas in kJ/kg·K. *T*_5_ is the exhaust gas to calorimeter inlet temperature in K. *T*_*a*_ is the ambient temperature in K.

An exhaust gas calorimeter is used for the measurement of heat carried by exhaust gases. It is a simple heat exchanger in which part of the heat of the exhaust gases is transferred to the circulating water. The hot gases are cooled by the water circulated in the calorimeter. It is assumed that the calorimeter is well insulated; there is no heat loss except by heat transfer from the exhaust gases to the circulating water and then for the calculation of *C*_*pe*_.

Heat lost by exhaust gases = heat gained by circulating water:

(10) $$\begin{array}{c} m_{ge}\times C_{pe}\times \left(T_{5}-T_{6}\right)=m_{\text{cw}}\times C_{pw}\left(T_{4}-T_{3}\right), \end{array}$$

where *m*_cw_ is the mass of cooling water passing through the calorimeter in Kg/sec. *T*_3_ is thecalorimeter water inlet in K. *T*_4_ is the calorimeter water outlet temp in K. *T*_5_ is the exhaust gas to calorimeter inlet temperature in K. *T*_6_ is the exhaust gas from calorimeter outlet temperature in K. *C*_*pe*_ is the specific heat of exhaust gases in kJ/kg K. *C*_*pw*_ is the specific heat of cooling water in kJ/kg K.

*(iv) Unaccounted Energy Losses *(*Q*_*u*_)* in kW*. A part of the heat is also lost by convection and radiation as well as by the leakage of gases. Part of the power developed inside the engine is also used to run the accessories as lubricating pump, cam shaft, and water circulating pump. This cannot be measured precisely and so this is known as unaccounted “losses.” This unaccounted heat energy is the difference between the heat supplied and the sum of heat equivalent of brake power + heat carried away by cooling water + heat carried away by the exhaust gases [16].

Therefore, unaccounted energy losses (*Q*_*u*_) in kW can be stated as

(11) $$\begin{array}{c} Q_{u}=Q_{s}-\left(Q_{\text{BP}}+Q_{\text{cw}}+Q_{\text{ex}}\right). \end{array}$$

### 2.7. Exergy Analysis

The performance of engine is analyzed in light of the 2nd law of thermodynamics, which narrates the quality of energy and determines the lost opportunities to do work. An exergy balance is the availability of fuel energy utilized in various ways which includes availability in shaft, cooling water, exhaust, and destructed. Exergy efficiency is the ratio between exergy in product to total exergy input [17].

**Figure 4:**
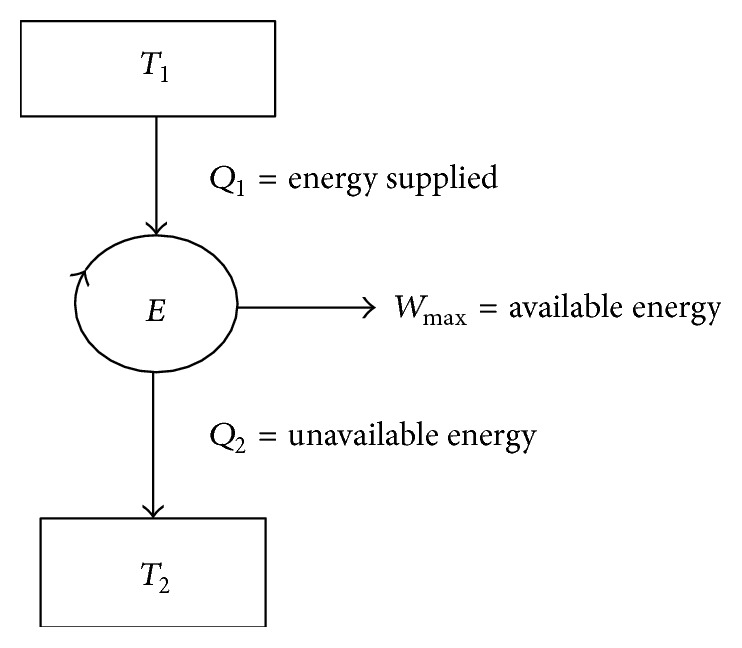
Available and unavailable energy in a cycle.

The available energy (A.E.) referred to a cycle is the maximum portion of energy which could be converted into useful work by ideal processes which reduces the system to a dead state. The minimum energy that has to be rejected to the sink by the second law is called the “Unavailable Energy (U.E.)” Available and unavailable energy in a cycle are shown in Figure 4.

The available energy refers to a diesel engine:

(12) Q1=A.E.+U.E.,Wmax⁡=A.E.=Q1−U.E.

#### 2.7.1. Exergy Balance

Exergy balance can be stated as the exergy change of a system during a process. It is equal to the difference between the net energy transfer through the system boundary and the energy destroyed within the system boundaries as a result of irreversibility. Exergy can be transferred to or from a system by heat work and mass transfer. The energy balance for any system undergoing any process can be expressed in the rate form is

(13) $$\begin{array}{c} {\dot{E}x}_{\text{in}}-{\dot{E}x}_{\text{out}}-{\dot{E}x}_{\text{destroyed}}=\frac{{dEx}_{\text{system}}}{dt}\;\left(\text{kW}\right) \end{array}$$

(14) ⟹Exheat−Exwork+Exmass,in−Exmass,out−Exdestroyed  =dExsystemdt,

where *Ex*_in_ − *Ex*_out_ is the rate of net energy transfer by heat, work, and mass. *Ex*_destroyed_ is the rate of energy destroyed:

(15) $$\begin{array}{c} {\dot{E}x}_{\text{destroyed}}=T_{0}s_{\text{gen}}\geq 0 \end{array}$$

for irreversible process *E*_destroyed_ > 0, and for reversible process *E*_destroyed_ = 0.

#### 2.7.2. Exergy Balance for Steady State Process

Common examples of control volume systems are turbine, heat transfer equipment, compressor, and so forth, which operate steadily. The amount of exergy entering a steady flow system (heat, work, and mass transfer) must be equal to the amount of exergy leaving the system plus the exergy destroyed.

In a steady flow system, [12] can be expressed as

(16) $$\begin{array}{c} {Ex}_{\text{heat}}-{Ex}_{\text{work}}+{Ex}_{\text{mass},\text{in}}-{Ex}_{\text{mass},\text{out}}-{Ex}_{\text{destroyed}}=0, \end{array}$$

(17) $$\begin{array}{c} ⟹\sum \left(1-\frac{T_{0}}{T}\right)\overset{\cdot }{Q}-\overset{\cdot }{W}+\overset{\cdot }{m}\left(e_{1}-e_{2}\right)-{\dot{E}x}_{\text{destroyed}}=0, \end{array}$$

(18) ⟹∑s1−T0TQ·−W·+∑inm˙·e−∑outm˙·e−E˙xdestroyed  =0,

where *T* is the absolute temperature at the location on the boundary where the heat transfer occurs. $\sum_{\text{in}}\dot{m}\cdot e-\sum_{\text{out}}\dot{m}\cdot e$ is the rate of exergy entering and leaving the control volume accompanying the fuel stream, respectively. ${\dot{E}x}_{\text{destroyed}}=T_{0}\cdot s_{\text{gen}}$, *s*_gen_ is the entropy generation.

Equation (18) is the rate of exergy change within the control volume during a process and is equal to the rate of net exergy transfer through the control volume boundary by heat, work, and mass flow minus the rate of exergy destruction within the boundaries of the control volume [10].

*e* is the flow exergy per unit mass and is defined as follows [19]:

(19) $$\begin{array}{c} e=e_{\text{tm}}+e_{\text{ch}}, \end{array}$$

where *e*_tm_ and *e*_ch_ are thermomechanical and chemical exergy, respectively:

(20) $$\begin{array}{c} e_{\text{tm}}=h-h_{0}-T_{0}\left(s-s_{0}\right), \end{array}$$

where *h* and *s* are flow enthalpy and flow entropy per unit mass at the relevant temperature and pressure, respectively, while *h*_0_, *s*_0_ stand for the corresponding values of these properties when the fluid comes to equilibrium with the reference environment.

#### 2.7.3. Exergies of the Liquid Fuels

The thermomechanical exergy of the fuel is zero [18]. The specific chemical exergy of liquid fuels can be evaluated on unit mass basis as (Kotas, 1995)

(21) efch=LHV1.0401+0.1728HC     +0.0432OC+0.2169SC      ×1−2.0628HC,

where H, C, O, and S are the mass fraction of hydrogen, carbon, oxygen, and sulphur, respectively.

In this study, it is assumed that the reference environment has a temperature (*T*_0_) of 298.15 K and a pressure of 1 atm. The reference environment is considered a mixture of perfect gases.

#### 2.7.4. Exergy of Exhaust Gas

The exhaust gas can be assumed as a mixture of ideal gases [20]. It is assumed that there is no water vapour in the combustion air. Then the thermomechanical exergy of the exhaust gas at the temperature *T*and pressure *P*, and containing *n* components *i*, can be obtained as follows.

The thermomechanical exergy of the exhaust gas is

(22) etm=∑i=1naih−iT−h−iT0−T0s−0T−s−0T0−R−ln⁡⁡PP0     ×s−0T−s−0T0−R−ln⁡⁡PP0,

where *a*_*i*_ is the coefficient of the component *i* in the reaction equation shown in (23), $\overset{-0}{s}$ is the absolute entropy at the standard pressure and in the exhaust gas, and $\overset{-}{R}$ is the universal gas constant (8.314 kJ/kmol·K).

The general form of reaction equation is (27):

(23) CxHy+aO2+3.76N2  ⟶bO2+cCO+dCO2+eCxHy+fN2+g  H2O

where *a*, *b*, *c*, *d*, and *e* are the coefficients of the component and C_*x*_H_*y*_ is the hydrocarbon. Thus, by applying conservation of mass principle to the carbon, hydrogen, and nitrogen, the unknown coefficients in (23) can be determined.

The chemical exergy of the exhaust gas is

(24) $$\begin{array}{c} e_{\text{ch}}=\overset{-}{R}\;T_{0}\overset{n}{\underset{i=1}{\sum }}a_{i}\ln\left(\frac{Y_{i}}{Y_{i}^{e}}\right), \end{array}$$

where *Y*_*i*_ is the molar ratio of the *i*th component in the exhaust gas and *Y*_*i*_^*e*^ is the molar ratio of the *i*th component in the reference environment. Furthermore, the reference environment is considered a mixture of perfect gases with the following composition on a molar basis: N_2_, 75.67%; O_2_, 20.35%; CO_2_, 0.0.3%; H_2_O, 3.12%; and other, 0.83% [18].

The thermomechanical and chemical exergy of the combustion air are ignored because the intake of air was very close to the reference state in all the test operation. Thus, the specific flow exergy of the exhaust gas per mole of fuel is the sum of the result of (22) and (24) [20].

#### 2.7.5. Exergy Rate from the Cooling Water to the Environment

Exergy rate from the cooling water to the environment is defined as the output heat rate from the engine to the environment through the cooling water of the engine [21]:

(25) $$\begin{array}{c} {Ex}_{\text{heat}}=\sum \left(1-\frac{T_{0}}{T_{cw}}\right)\dot{Q}, \end{array}$$

where *T*_0_ is the reference (dead) state temperature and *T*_cw_ is the cooling water temperature.

*Exergy Balance Calculations for the Present Experiment*. In the present experimental analysis, the availability of fuel supplied (*A*_in_) is converted into shaft availability (*A*_*s*_), cooling water availability (*A*_cw_), exhaust gas availability (*A*_*e*_), and destructed availability.

*Availability of Fuel *(*A*_*in*_)* in kW*. The specific chemical exergy of liquid fuel on a unit mass basis can be evaluated as

(26) A˙in=LCVfHC ×1.0401+0.1728HC+0.0432OC   +0.2169SC×1−2.0268HC

where H, C, O, and S are the mass fraction of hydrogen, carbon, oxygen, and sulphur [22].

(i) Shaft availability (*A*_*s*_) = brake power of the engine in kW.
(ii) Cooling water availability (*A*_cw_) in kW is
  (27) $$\begin{array}{c} A_{\text{cw}}=Q_{\text{cw}}-\left[m_{we}\times C_{pw}\times T_{a}\times \ln\left(\frac{T_{2}}{T_{1}}\right)\right], \end{array}$$
  where *m*_*we*_ is the mass of cooling water circulated through the cooling jacket, kg/s. *C*_*pw*_ is the specific heat of water kJ/kg K. *T*_1_ is the inlet water temperature passing through the cooling jacket, K. *T*_2_ is the outlet water temperature of cooling jacket, K. *T*_*a*_ is the ambient temperature, K.
(iii) Availability of exhaust gas (*A*_ex_), in kW is
  (28) Aex=Qex−mge×TaT5Ta ×Cpeln⁡⁡T5Ta−Reln⁡⁡PePa+ech,
  where *R*_*e*_ is the specific gas constant of the exhaust gas in kJ/kg K. *P*_*a*_ is the ambient pressure, N/m^2^. *P*_*e*_ is the final pressure, N/m^2^. *T*_*a*_ is the ambient temperature, K. *m*_*ge*_ is the mass of exhaust gas, kg/s. *T*_5_ is the exhaust gas to calorimeter inlet temperature, K.
(iv) Destructed availability (*A*_*d*_) in kW is
  (29) $$\begin{array}{c} A_{d}=A_{\text{in}}-\left(A_{s}+A_{\text{cw}}+A_{\text{ex}}\right) \end{array}$$
  and exergy efficiency (*η*_*A*_) in %:
  (30) $$\begin{array}{c} \eta_{A}=\left[1-\left(\frac{A_{d}}{A_{\text{in}}}\right)\right]\times 100. \end{array}$$

Chemical composition of Mahua oil and biodiesel is shown in Table 5.

The molecular formula of biodiesel is obtained by considering

(31) No.  of  any  element  in  biodiesel  =No.  of  that  element  in  compoud    ∗%  of  that  compoud×Total%−1.

Molecular formula of B20 is calculated as follows.

Numbers of C, H, O, and S atoms are calculated by considering 80% of diesel (C_12_ H_26_S_0.0024_) and 20% of Mahua biodiesel (C_18.63_H_35.87_O_2_).

Based on the above chemical composition, the molecular formula of B20 is evaluated and shown in Table 6.

Mass fraction ratio of H, C, and O of diesel and B20 is calculated and shown in Table 7.

## 3. Result and Discussion

The biodiesel was blended as per the requirement and various properties were found out. The important properties of various blends of MOME were compared with diesel. The performance and characteristics of different blends of MOME were also compared with diesel by conducting various experiments on the above said engine.

### 3.1. Calorific Values

Calorific value implies the heat produced by the fuel to do the useful work within the engine. Heating value is commonly determined by use of a bomb calorimeter. The heat of combustion of the fuel samples was calculated with the help of equation given below:

(32) $$\begin{array}{c} H_{c}=\frac{W_{c}\times \Delta T}{m_{s}}, \end{array}$$

where *H*_*c*_ is the heat of combustion of the fuel sample, kJ/kg; *W*_*c*_ is the water equivalent of the calorimeter assembly, kJ/°C;* ΔT* is the rise in temperature, °C; *m*_*s*_ is the mass of burnt sample, kg.

The calorific values of different blends of B20, B30, B40, and B100 were 41.13, 41.00, 40.00, and 37.00 Mj/Kg, respectively. It indicates that the calorific value of all the blends was lower than diesel and as the blend increases the calorific value decreases.

### 3.2. Specific Gravity

The specific gravity of a liquid is the ratio of its specific weight to that of pure water at a std. temperature. Specific gravity is determined by Pycnometer method:

(33) Specific  gravity  =weight  of  bottle  and  sample    −weight  of  bottle   ×weight  of  water  at  a  std.temperature−1.

The specific gravity of B20, B30, B40, and B100 was 0.865, 0.868, 0.875, and 0.88, respectively. The specific gravity of B20 is 1.02 times dense as diesel. The specific gravity decreases as the temperature increases. A higher specific gravity indicated higher energy content in the fuel.

### 3.3. Kinematic Viscosity

Viscosity is a measure of the internal resistance to motion of a fluid and is mainly due to the forces of cohesion between the fluid molecules. For determination of kinematic viscosity in the laboratory, kinematic viscometer is used:

(34) $$\begin{array}{c} \text{Kinematic}\;\text{Viscosity}=C*t, \end{array}$$

where *t* is the flow time, s;* C* is the calibration constant of the viscometer, 0.0336 cSt/s.

The kinematic viscosity of B20, B30, B40, and B100 was 4.35, 4.45, 4.52, and 4.98 in mm^2^/sec, respectively. The kinematic viscosity of the MOME reduced from 37.18 of crude oil to 4.98 after transesterification, which results in better atomization without preheating. It further reduced with increase in blending with diesel.

### 3.4. Engine Performance

#### 3.4.1. Brake Specific Fuel Consumption

BSFC is a measure of fuel efficiency in a shaft reciprocating engine. It is the rate of fuel consumption per hour divided by the power produced.

**Figure 5:**
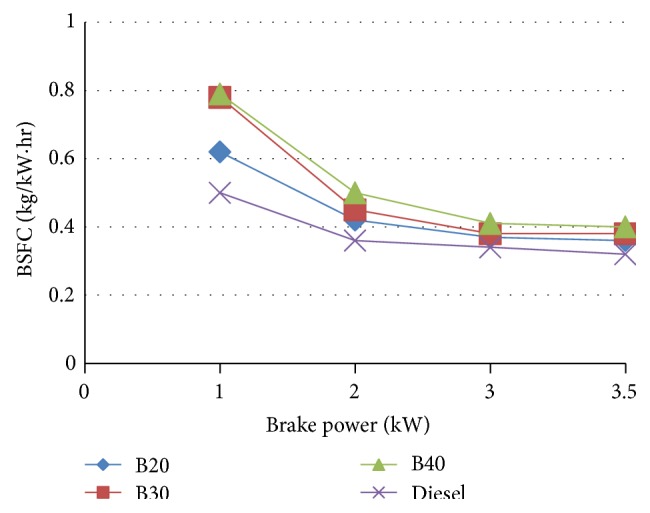
Brake specific fuel consumption versus brake power.

Figure 5 is the comparison graph of BSFC of different blends of biodiesel at different loads. The graph indicates that BSFC increases with the increase in blends of biodiesel. For B20, BSFC is increased by 24% at minimum load and 5.71% at maximum load. This increase is due to poor atomization of fuel, lower calorific value, and higher viscosity. Thus, at higher load, B20 approach is very close to the diesel.

#### 3.4.2. Brake Thermal Efficiency

Brake thermal efficiency is the ratio of brake power output to power input, that is, heat equivalent to one Kw.Hr. divided by heat in fuel per BP hour.

**Figure 6:**
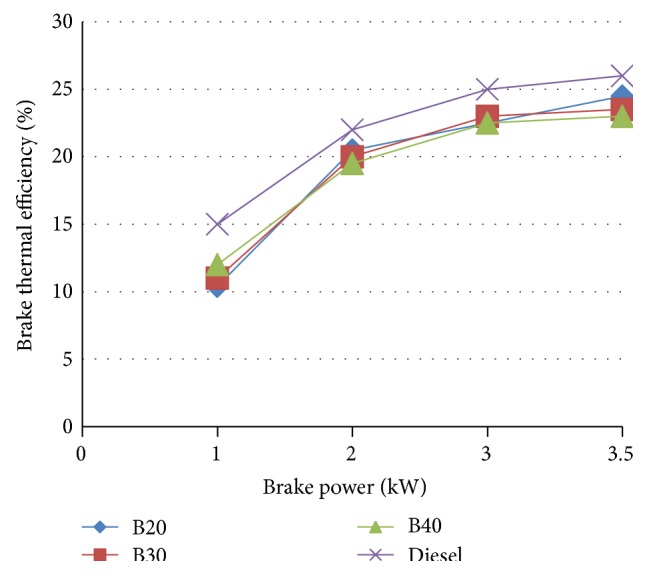
Brake thermal efficiency versus brake power.

Figure 6 shows the variation of BTE with various blends and diesel. The reduction in BTE with biodiesel blends at higher loads was due to higher viscosity, poor atomization, and low calorific value. At higher load, the BTE increases for B20 and B30 blends. B20 is found to have the maximum brake thermal efficiency at higher loads among the blends.

### 3.5. Emission Profile

#### 3.5.1. CO_2_ Emission

**Figure 7:**
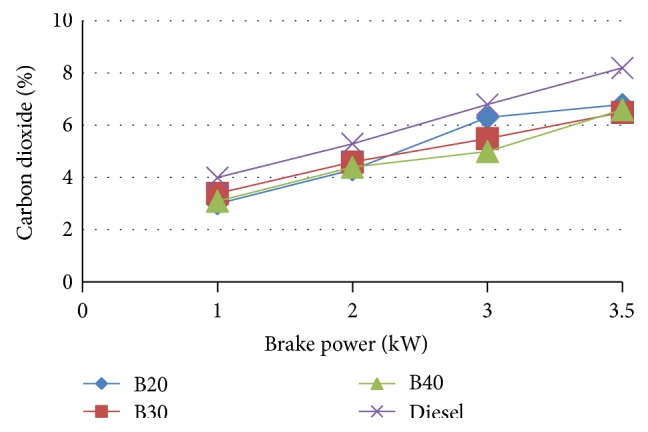
Variation of carbon dioxide with brake power.

The variation of CO_2_ with respect to brake power for different blends of MOME is shown in Figure 7. The composition of carbon dioxide is found more for diesel compared to various blends of MOME. The emission of CO_2_ trend is an increasing trend as load increases. This rising trend may be due to more fuel consumption as load increases. As compared to diesel, the blends emissions are found to be less.

#### 3.5.2. NO_*x*_ Emission

**Figure 8:**
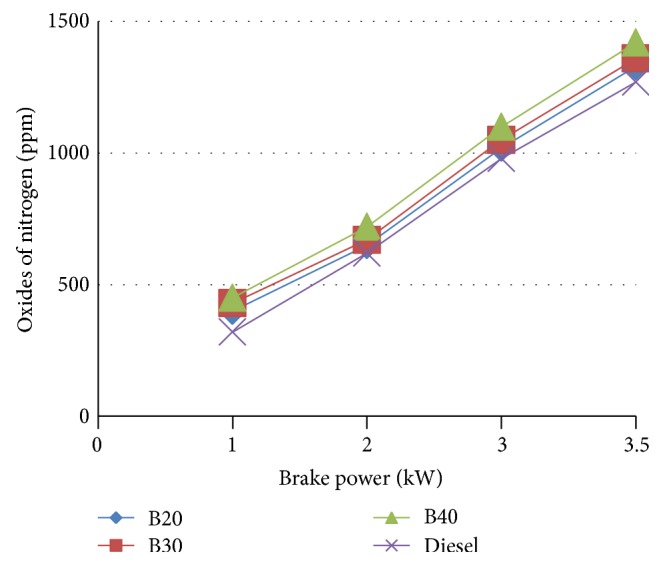
Variation of oxides of nitrogen with brake power.

Figure 8 indicates the variation of NO_*x*_ concentration with engine load for various blends of MOME. When compared to diesel, the blends show an increasing trend with respect to load. As the temperature of exhaust gas increases at higher loads the NO_*x*_ composition increases.

#### 3.5.3. Hydrocarbon Emission

**Figure 9:**
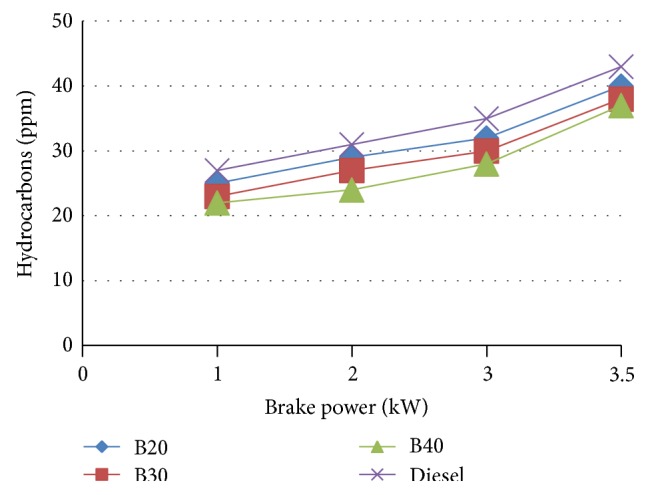
Variation of hydrocarbons with brake power.

Figure 9 indicates the variation of Hydrocarbons concentration with engine load for various blends of MOME. It is observed from the graph that Mahua ester based fuel emission rate of hydrocarbon is less than diesel. As the blends increases, the emission of HC decreases. This indicates there is a complete combustion of fuel. This may be due to presence of more oxygen in the fuel.

#### 3.5.4. Carbon Monoxide Emission

**Figure 10:**
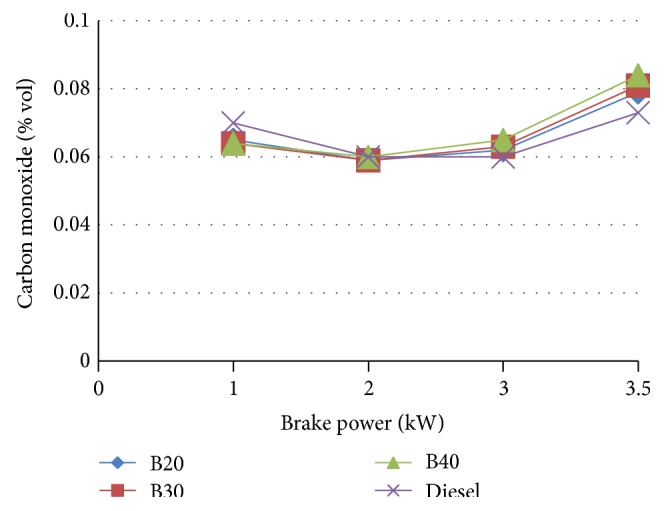
Variation of carbon monoxide with brake power.

Figure 10 shows the variation of carbon monoxide with brake power. It was observed that as the load increases the emission also increases. At low and medium loads the carbon monoxide emissions of all blends are very close. As the load increases the emission of blends increases compared to diesel.

As per the performance and emission profile are concerned, it is observed that B20 is found to be most suitable as a fuel in I.C engine. Many of the authors recommended that blends of up to 20% biodiesel mixed with petroleum diesel fuels can be used in nearly all diesel equipment and are compatible with most storage and distribution equipment [4, 22–24]. Keeping this factor in mind, we consider to proceed to energy and exergy analysis for B20 blends and compare the results with diesel fuel.

### 3.6. Energy Analysis

An energy analysis sheet shown in Table 8 is an account of energy supplied and utilized by using diesel and B20. For the calculation purpose specific heat of water is taken as 4.18 kJ/kg·K and that of exhaust gas is based on the heat lost by exhaust gasses which is equal to heat gained by circulating water.

**Figure 11:**
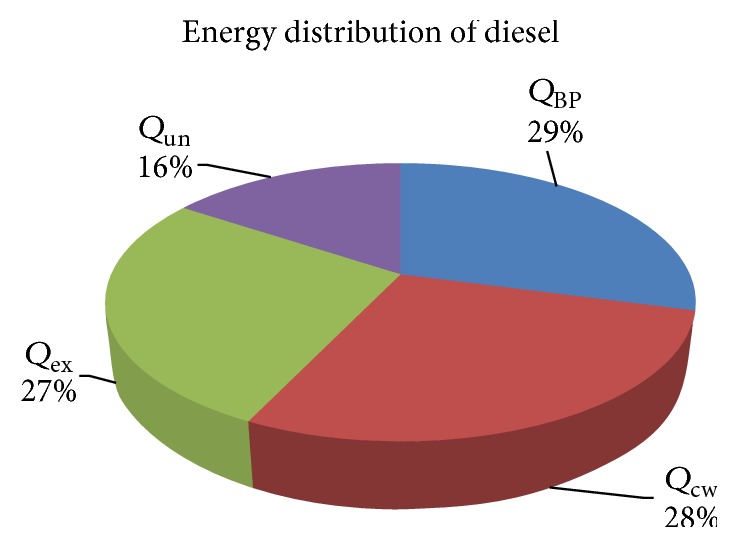
Energy distribution of diesel.

**Figure 12:**
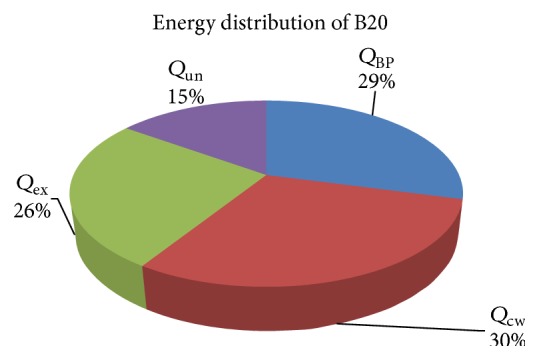
Energy distribution of B20.

Energy distribution of diesel and B20 is shown in Figures 11 and 12 in graphical format.

**Figure 13:**
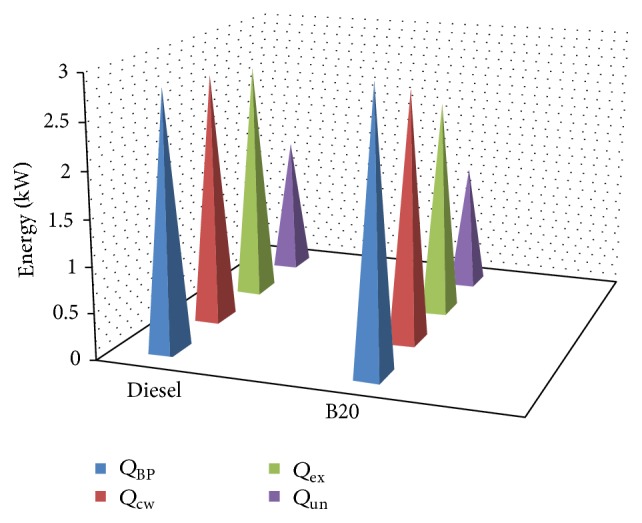
Comparison of energy distribution of diesel and B20.

Comparison of energy distribution of diesel and B20 is shown in Figure 13.

### 3.7. Exergy Analysis

By sighting the exergy analysis equations, the distributions of exergy per unit time for diesel and B20 are listed in (Table 9).

**Figure 14:**
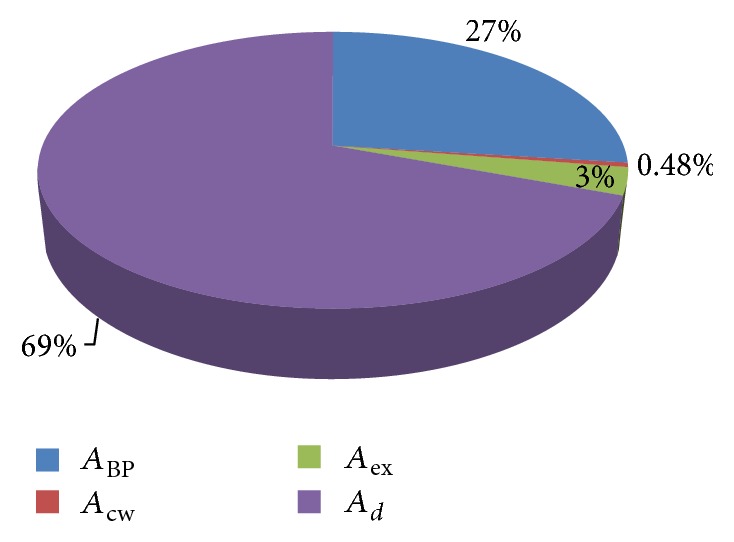
Exergy distribution of diesel.

**Figure 15:**
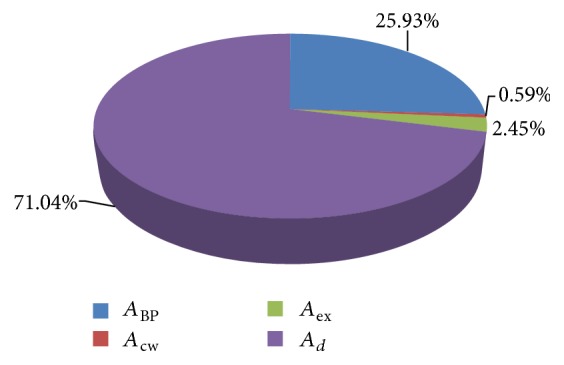
Exergy distribution of B20.

**Figure 16:**
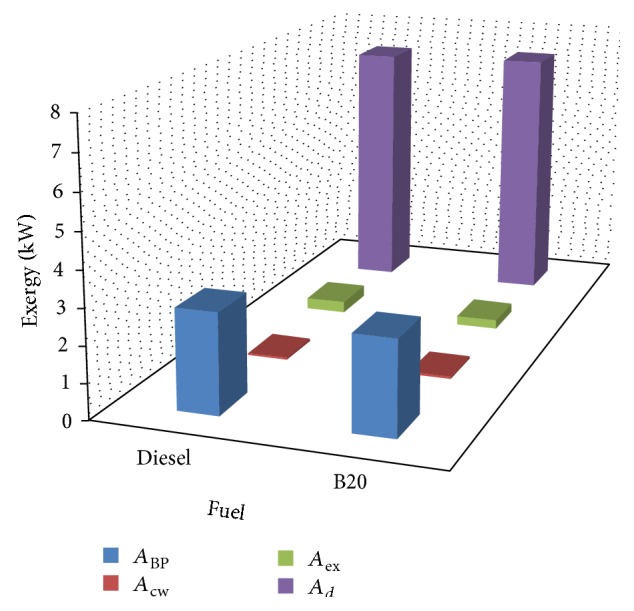
Comparison of exergy distribution for diesel and B20.

Graphical representations of exergy distribution of diesel and B20 are shown in Figures 14 and 15, respectively. Comparison of exergy distribution for diesel and B20 is shown in Figure 16.

## 4. Conclusion

The major conclusions were drawn on the basis of the engine tests which were carried out in a 3.74 kW diesel engine in the engine lab. Energy analysis is based on the 1st law of thermodynamics.

For B20, BSFC is increased by a marginal value of 5.71% at maximum load compared to diesel. The brake thermal efficiency of diesel is more than biodiesel but at higher load B20 approach is very close to the diesel. B20 is found to have the maximum brake thermal efficiency at higher loads among the blends approaching that of diesel. A marginal increase in NO_*x*_ emission was noted in blended oils. However, CO_2_, HC emission is decreased. At full load, the carbon monoxide emissions of the fuels increase. For B20 at higher loads the emission rate is close to that of diesel.

From energy analysis, it was observed that the fuel energy input as well as energy for BP and energy flown through exhaust gases and unaccounted losses were more in case of diesel than B20. The energy efficiency of diesel was 28% while the total losses were 72%. In case of B20, the efficiency was higher (29%) and lower losses were observed than that of diesel. The fuel energy input of diesel is 6.25% more than B20 due to high heating value of diesel. The exergy efficiency of diesel and B20 was 30.66% and 28.96%, respectively.

The input availability of diesel fuel is 1.46% more than B20. Shaft availability of diesel is more than that of B20. Exhaust gas availability of diesel is more than that of B20. The system inefficiency is the destructed availability which is found more in case of B20.

It can be concluded that B20 fuel shows almost similar energetic and exergetic performance value with diesel.

All the tests are conducted by the engine without making any engine modification. From the above observation, B20 blend of Mahua biodiesel can be recommended for use in diesel engine as per as engine performance and emission profile are concerned. Also B20 shows almost similar energetic performance. So citing the above conclusion B20 can be a substitute for diesel.

Mahua flower is also fermented to produce the alcoholic drink, country liquor whose consumption allows many health related problems. Production of MOME from Mahua can be a solution which will not only decrease the production of country liquor but also improve socioeconomic condition.

## Figures and Tables

**Table 1:** Fatty acid profile of Mahua oil [1].

Fatty acid	Systemic name	Formula	Structure	Wt. %
Palmitic	Hexadecanoic	C_16_H_32_O_2_	16:0	16–28.2
Stearic	Octadecanoic	C_18_H_36_O_2_	18:0	20–25.1
Arachidic	Eicosanoic	C_20_H_40_O_2_	20:0	0.0–3.3
Oleic	Cis-9-Octadecenoic	C_18_H_34_O_2_	18:1	41.0–51.0
Linoleic	Cis-9,cis-12-Octadecadienoic	C_18_H_32_O_2_	18:2	8.9–13.7

**Table 2:** Acid values of Mahua oil.

Mahua oil	Acid values
Crude oil	34
After esterification	2.3
After transesterification	0.45

**Table 3:** Fuel properties of Mahua oil, MOME, and diesel.

Fuel properties	Mahua oil	MOME	Diesel
Calorific value (Mj/Kg)	38.86	37.0	45.34
Specific gravity	0.904	0.880	0.842
Kinematic viscosity at 40°C (cSt)	37.18	4.98	2.44
Flash point (°C)	238	208	63.0
Fire point (°C)	244	240	68.0
Carbon residue (%)	0.42	0.2	0.034

**Table 4:** Engine specifications.

Engine parameters	Specifications
Manufacturer	Kirloskar
Number of stroke	4
Number of cylinder	Single
Type	Vertical, constant speed, and direct injection
Compression ratio	16.5 : 1
Rated power	3.74 Kw
Speed	1500 r.p.m.
Bore × stroke (mm)	80 × 110
Cooling	Water cooled
Lubrication used	20W40

**Table 5:** Chemical composition of Mahua oil and biodiesel.

Fatty acid Mol. formula	Methyl easters Mol. formula	% Age	Mol. Wt. of Mahua biodiesel	Mol. formula of Mahua biodiesel
C_16_H_32_O_2 _	C_17_H_34_O_2_	24.5	291.13 g/mol	C_18.63_H_35.87_O_2_
C_18_H_36_O_2 _	C_19_H_38_O_2_	22.5		
C_20_H_40_O_2 _	C_21_H_42_O_2_	1.5		
C_18_H_34_O_2_	C_19_H_36_O_2_	37.5		
C_18_H_32_O_2 _	C_19_H_34_O_2_	14.3		

**Table 6:** Molecular formula of diesel and B20.

Fuel	Molecular formula
Diesel	C_12_H_26_S_0.0024_
B20	C_13.32_H_27.37_O_0.4_S_0.00192 _

**Table 7:** Mass fraction ratio of H, C, and O of diesel and B20.

Elements	Diesel	B20
H/C	0.182	0.17
O/C	—	0.03
S/C	0.00047	0.003

**Table 8:** Energy balance sheet for diesel and B20.

Fuel energy supplied (Kw)		Energy expenditure (Kw)	Diesel (Kw)	B20 (Kw)
Diesel	B20	Energy in brake power (*Q*_BP_)	2.80	2.65
9.68	9.11	Energy carried by cooling water (*Q*_cw_)	2.73	2.73
		Energy carried away by exhaust gasses (*Q*_ex_)	2.64	2.36
		Unaccounted energy loss (*Q*_un_)	1.51	1.37

**Table 9:** Exergy balance sheet of diesel and B20.

Exergy of fuel (kW)		Distribution of exergy (kW)	Diesel (Kw)	B20 (kW)
Diesel	B20	Exergy in brake power (*A*_bp_)	2.80	2.65
*A*_in_ = 10.37	*A*_in_ = 10.22	Exergy in cooling water (*A*_cw_)	0.05	0.06
		Exergy of exhaust gases (*A*_ex_)	0.33	0.25
		Destructed exergy (*A*_*d*_)	7.19	7.26

## Nomenclature

$\overset{\cdot }{E}$: Rate of net energy transfer, kW
$\dot{Q}$: Heat transfer, kW
$\dot{W}$: Work done, kW
*h*: Enthalpy, kJ/kg
*V*: Velocity, m/s
*Z*: Elevation, m
*q*: Heat transfer per unit mass, kJ/kg
*w*: Work done per unit mass, kJ/kg
*T*: Corresponding temperature, K
*C*_*p*_: Specific heat at constant pressure, kJ/kg K
*Q*_BP_: Heat equivalent of brake power, kW
*Q*_cw_: Heat carried away by cooling water, kW
*Q*_ex_: Heat carried away by exhaust gases, kW
*Q*_*u*_: Unaccounted energy losses, kW
BP: Brake power, kW
*m*_*f*_: Mass of fuel supplied, kg/s
*m*_*we*_: Mass of cooling water circulated through the cooling jacket, kg/s
*m*_*cw*_: Mass of cooling water passing through the calorimeter, kg/s
*m*_*ge*_: Mass of exhaust gases (*mf* + *ma*), kg/s
L.C.V.: Lower calorific value, kJ/kg
*N*: Crank revolution per second
*T*_*e*_: Torque developed, Nm
*C*_*pw*_: Specific heat of water, kJ/kg K
*C*_*pe*_: Specific heat of exhaust gas, kJ/kg K
*T*_*a*_: Ambient temperature, K
A.E.: Available energy, kW
U.E.: Unavailable energy, kW
*e*: Flow exergy per unit mass
*e*_tm_: Thermomechanical exergy
*e*_ch_: Chemical exergy
*e*_*f*_^ch^: Specific chemical exergy
*a*_*i*_: Coefficient of the component *i*
$\overset{-}{R}$: Universal gas constant, kJ/kmol-K
*Y*_*i*_: Molar ratio of the *i*th component in the exhaust gas
*Y*_*i*_^*e*^: Molar ratio of the *i*th component in the reference environment
*A*_in_: Input availability, kW
*A*_cw_: Cooling water availability, kW
*A*_ex_: Exhaust gas availability, kW
*A*_*d*_: Destructed availability, kW
*η*_*A*_: Exergy efficiency
*s*: Entropy, kJ/kg K.
